# The relationship and clinical significance of lactylation modification in digestive system tumors

**DOI:** 10.1186/s12935-024-03429-8

**Published:** 2024-07-15

**Authors:** Gang Wang, Xiaosu Zou, Qicong Chen, Wenqian Nong, Weiwei Miao, Honglin Luo, Shenhong Qu

**Affiliations:** 1 Institute of Oncology, Guangxi Academy of Medical Sciences, Nanning, 530021 Guangxi China; 2 Department of Otolaryngology & Head and Neck, People’s Hospital of Guangxi Zhuang Autonomous Region, Nanning, 530021 Guangxi China

**Keywords:** Lactate, Lactylation, Digestive system tumors, Epigenetics, Tumor microenvironment

## Abstract

Lactylation, an emerging post-translational modification, plays a pivotal role in the initiation and progression of digestive system tumors. This study presents a comprehensive review of lactylation in digestive system tumors, underscoring its critical involvement in tumor development and progression. By focusing on metabolic reprogramming, modulation of the tumor microenvironment, and the molecular mechanisms regulating tumor progression, the potential of targeting lactylation as a therapeutic strategy is highlighted. The research reveals that lactylation participates in gene expression regulation and cell signaling by affecting the post-translational states of histones and non-histone proteins, thereby influencing metabolic pathways and immune evasion mechanisms in tumor cells. Furthermore, this study assesses the feasibility of lactylation as a therapeutic target, providing insights for clinical treatment of gastrointestinal cancers. Future research should concentrate on elucidating the mechanisms of lactylation, developing efficient lactylation inhibitors, and validating their therapeutic efficacy in clinical trials, which could transform current cancer treatment and immunotherapy approaches. In summary, this review emphasizes the crucial role of lactylation in tumorigenesis and progression through a detailed analysis of its molecular mechanisms and clinical significance.

## Introduction

Malignant tumors of the digestive system constitute a group of cancer types that develop in the digestive tract and its related organs, including esophageal cancer (EC), gastric cancer (GC), colorectal cancer (CRC), hepatocellular carcinoma (HCC), and pancreatic cancer (PC), among others. The occurrence and progression of these malignant tumors involve a complex and multifactorial process. Globally, malignant tumors of the digestive system represent one of the major types of cancer, with epidemiological characteristics showing significant variations among different regions and populations. Particularly high incidences of EC have been observed in certain regions of East Asia and Africa [1]. Regions with high incidence rates of GC include East Asia, Eastern Europe, and South America, with a higher incidence generally observed in males compared to females [2]. In contrast, the incidence of CRC is relatively high in developed countries [3]. Approximately 80% of HCC cases worldwide occur in developing countries such as those in Sub-Saharan Africa and East Asia, closely associated with hepatitis B virus infection and aflatoxin B1 intake [4]. PC primarily affects the East Asian region, with a relatively low incidence but high mortality rate and poor prognosis, often remaining undetected until the advanced stages [5]. PC ranks fourth in the 5-year survival rate for cancer-related deaths in Europe, with an estimated projection that by 2030, it will become the second leading cause of cancer-related mortality [6, 7].

It is well known that metabolic reprogramming and epigenetic reshaping are closely related and mutually regulated, serving as one of the hallmarks of cancer. Epigenetic dysregulation contributes to the initiation and progression of tumors, promoting dynamic gene expression patterns and facilitating tumor evolution and therapy adaptation [8]. Epigenetics primarily regulates gene expression through processes such as methylation and histone modification, without altering the genetic sequence, and these processes are crucial for maintaining normal cellular function [9]. Increasing evidence suggests that aberrations in epigenetic processes lead to the inactivation of tumor suppressor genes or the activation of oncogenes, thereby promoting malignant transformation of cells [10, 11]. Among the epigenetic regulatory mechanisms, histone modification is particularly significant. Histones are basic proteins that interact with DNA and constitute the main protein component of chromatin. Different types of histone modifications, such as acetylation, methylation, and ubiquitination, can influence chromatin structure, thereby regulating gene transcription. In human tumors, the abnormal activities of many histone-modifying enzymes have been found to be involved in tumorigenesis. For instance, the overexpression of histone deacetylases is associated with the malignant biological behavior of tumors, and the overexpression of histone methyltransferase EZH2 can promote tumor proliferation and metastasis [12, 13]. Furthermore, non-coding RNAs can also mediate changes in histone modifications [14].

In recent years, a novel type of histone modification, known as histone lysine lactylation (Kla), has been discovered, utilizing the cellular metabolite lactate as a substrate for modification. Lactate has long been regarded as a metabolic waste product, but recent research indicates that it cannot only be recycled by tumor tissues to provide energy but can also serve as a modifying substrate mediating lysine lactylation of histones [15]. Tumor cells, in order to sustain their rapid proliferation, typically undergo metabolic reprogramming, with one of the most characteristic changes being enhanced glycolysis. Glycolysis is a glucose breakdown pathway that is less efficient but faster, providing sufficient energy for cells in low oxygen environments. In the tumor microenvironment (TME), cancer cell glycolysis is particularly active, even in conditions of high oxygen concentration. This phenomenon of lactate production under aerobic conditions is known as the “Warburg effect”. The main purpose of enhancing glycolysis in tumor cells is to acquire more intermediate metabolites, which can be utilized for the synthesis of lipids, nucleotides, and other biomolecules required for tumor proliferation. Meanwhile, lactate produced from glycolysis can drive extracellular matrix acidification, altering the microenvironment, which is unfavorable for the growth of normal cells but promotes tumor invasion and metastasis [16, 17]. Additionally, tumor-associated macrophages can also absorb lactate, further promoting their polarization toward the tumor-associated M2 phenotype [18]. Recent studies have found that lactate, as the final product of glycolysis, not only serves as a metabolic waste but also participates in gene expression regulation through pathways such as inducing histone lactylation. Lactate-mediated epigenetic changes are considered to be a critical link in how cellular metabolic status influences gene expression. Elevated levels of histone lactylation have been observed in multiple malignant tumors, suggesting its potential involvement in tumor development. However, the mechanisms and functions of this novel modification are currently poorly understood. A deeper understanding of the importance of epigenetic regulation, especially histone modification, in tumor development will contribute to further comprehension of the role of metabolic reprogramming in tumor initiation and progression, as well as the molecular mechanisms underlying tumorigenesis. This will facilitate the development of therapeutic strategies targeting the regulation of tumor cell metabolism.

To gain a comprehensive understanding of lactylation mechanisms, it is critical to focus on the roles of tumor suppressor genes and oncogenes in targeted pathways. The association between lactylation and the m6A reader protein YTHDF2 has been elucidated. YTHDF2 recognizes m6A-modified PER1 and TP53 mRNAs, promoting their degradation and thereby accelerating tumorigenesis [19]. Additionally, the link between lactylation and VHL gene inactivation has been shown to play a pivotal role in tumor progression. Inactivation of VHL leads to increased levels of histone lactylation, which subsequently activates the PDGFRβ signaling pathway. This reaction forms a positive feedback mechanism that accelerates tumor advancement [20]. In the TME, lactylation modifies the activity of tumor-associated macrophages (TAMs), thereby diminishing their anti-tumor effects. Inhibition of lactylation restore the phagocytic activity of TAMs, enhancing their anti-tumor capacity [21]. KRAS mutation-activated circATXN7 promotes tumor immune evasion by affecting T cell sensitivity, and lactylation plays an activating role in circATXN7, influencing the fate and function of T cells [22]. Moreover, LKB1 suppresses histone H4 lactylation, altering the activity of the transcription factor Sp1, which subsequently inhibits telomerase activity, leading to tumor cell senescence [23]. Lactylation has also been linked to proteasome inhibitor resistance. MUC20 inhibits the lactylation of MET, thereby reducing tumor cell resistance to proteasome inhibitors, indicating a role for lactylation in the development of drug resistance in tumor cells [24]. Furthermore, lactylation enhances SLUG expression by inhibiting PTEN transcriptional activity, which subsequently suppresses autophagy and affects cell function and tissue repair [25]. In summary, lactylation not only impacts the expression and function of oncogenes but also involves the regulation of the tumor immune microenvironment, tumor cell metabolism, and the formation of drug resistance and autophagy. By interacting with tumor-related genes, lactylation regulates gene expression and influences tumor cell proliferation, invasion, metabolism, and immune evasion. Research on lactylation, combined with the understanding of genes associated with tumors, provides valuable insights into the molecular mechanisms underlying digestive system tumors.

## Lactate metabolism and protein lactylation

### Lactate production and function

**Fig. 1:**
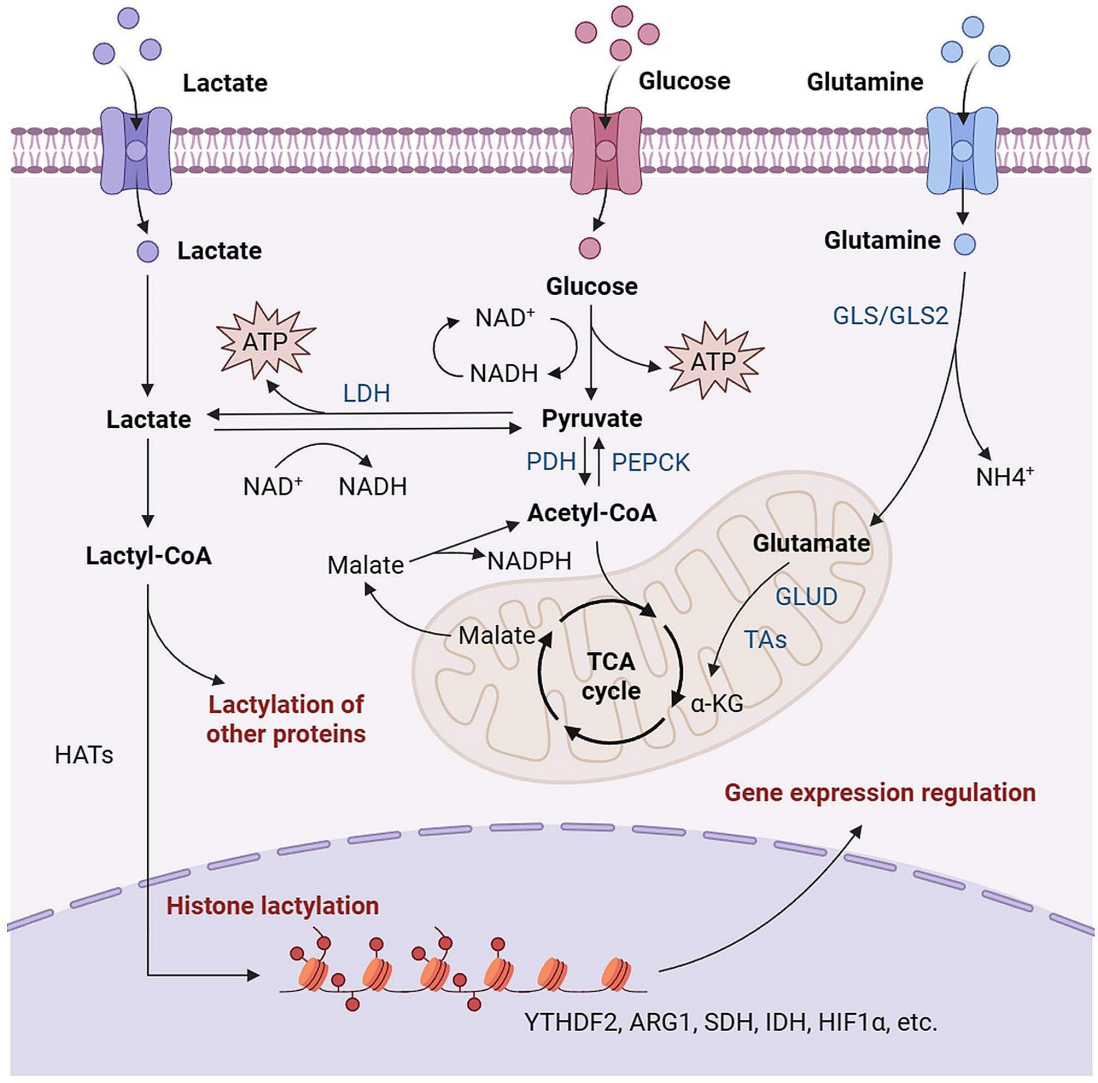
Production of lactate and lactylation. When cellular oxygen demand does not exceed the supply, glucose undergoes glycolysis, producing pyruvate, which enters the mitochondria and is oxidized to CO2 and H2O through the TCA cycle. However, under anaerobic or low-oxygen conditions inhibiting the TCA cycle, in order to sustain glycolysis, pyruvate is reduced to lactate in the cytoplasm by LDH. Specifically, glucose in the cytoplasm undergoes a series of classical catalytic reactions to convert into pyruvate. Pyruvate, instead of entering the mitochondria for oxidation, is directly reduced to lactate by LDH. Additionally, glutamine entering the cytoplasm is converted to glutamate through glutaminase. Then, glutamate is transformed into α-ketoglutaric acid through glutamate dehydrogenase and enters the TCA cycle. In this cycle, carbon derived from glutamine is converted to oxaloacetate, then transformed into malate and exits the mitochondria. Subsequently, through cytoplasmic malate dehydrogenase, it is converted to NADPH and pyruvate, further reduced to lactate by LDH. Lactate metabolism in cells involves two pathways. Firstly, lactate is oxidized to pyruvate, which enters the TCA cycle after conversion by pyruvate dehydrogenase. On the other hand, lactate can be converted to lactyl-CoA, which is catalyzed by histone acetyltransferase and participates in histone Kla. Simultaneously, lactyl-CoA is also involved in non-histone Kla. LDH: lactate dehydrogenase. PDH: pyruvate dehydrogenase. PEPCK: phosphoenolpyruvate carboxykinase. GLS: glutaminase. GLUD: glutamate dehydrogenase. TAs: aspartate or alanine transaminase. α-KG: α-ketoglutaric acid. HATs: histone acetyltransferases. The figure was created using Biorender.com

Lactate is the end product of the glycolytic pathway, generated under both normal physiological and pathological conditions in the human body. When cellular demand for oxygen does not exceed supply, glucose undergoes glycolysis to yield pyruvate, which then enters the mitochondria and is oxidized to CO2 and H2O through the tricarboxylic acid cycle. However, under anaerobic or low oxygen conditions such as during intense exercise or infection, to sustain the continuation of glycolysis, pyruvate is reduced to lactate by lactate dehydrogenase (LDH) in the cytoplasm. Specifically, glucose in the cytoplasm undergoes a series of classical catalytic reactions to convert into pyruvate, which does not enter the mitochondria for oxidation but is directly reduced to lactate by LDH. In addition to glycolysis, the degradation of glutamine is another source of lactate in cancer cells [26]. Glutamine entering the cytoplasm is converted to glutamate by glutaminase. Subsequently, glutamate is transformed into α-ketoglutarate by glutamate dehydrogenase, among other enzymes, and enters the tricarboxylic acid cycle. In this cycle, the carbon derived from glutamine is converted into oxaloacetate, which then becomes malate and exits the mitochondria. It is then converted into NADPH and pyruvate through cytoplasmic malic enzyme (Fig. 1). This represents a minor source of lactate production in cancer cells. Elevated serum lactate can lead to lactic acidosis, thus lactate needs to be rapidly metabolized from tissues and cells through the irreversible removal of lactate by LDH [27, 28]. The systemic balance between glycolysis and pyruvate dehydrogenase may be a critical determinant of lactate levels. Furthermore, lactate accumulation can activate gluconeogenesis in liver and skeletal muscle cells, converting lactate into glucose and releasing it into the bloodstream to drive additional glucose consumption during energy expenditure [29].

Lactate serves various functions in biological processes. It is no longer regarded solely as a metabolic waste but rather as a vital energy substrate and signaling molecule. Lactate is a principal raw material in the tricarboxylic acid cycle. For instance, circulating lactate acts as a supplementary source to meet the brain’s energy demands during glucose insufficiency, fulfilling the brain’s excitatory activities [30]. Other studies indicate that lactate is a major contributor to the activity of orexin neurons, rather than glucose [31, 32]. In tumor research, lactate’ contribution to the tricarboxylic acid cycle in pancreatic cancer exceeds that of glucose [33]. Secondly, lactate serves as an intercellular or intertissue redox signaling molecule. Oxidation reactions lead to electron release, with the oxidized nicotinamide adenine dinucleotide (NAD or NADP) receiving these electrons and being reduced to NADH or NADPH, which then get re-oxidized to release electrons during mitochondrial respiration or lactate fermentation, thus maintaining the intracellular redox balance [34]. Imbalance in redox reactions is detrimental; an overly oxidative state can generate more reactive species, accelerating cellular aging. Conversely, under a highly reducing state, cells have significantly diminished capacity to prevent oxidative stress and inhibit glycolysis. Given the interconversion of lactate and pyruvate, the NAD / NADH ratio in the cell alters during this conversion, with lactate providing suitable buffering to prevent excessive oxidative or reductive reactions. In summary, lactate is a significant contributor to the redox balance, stabilizing the cellular redox state by regulating other forms of energy metabolism when the ratio of oxidized to reduced coenzymes is imbalanced. Thirdly, lactate is involved in regulating fatty acid metabolism. Fatty acid synthesis is indispensable for cell membrane structure and function, energy storage, and signal transduction [35]. Under inflammatory conditions, lactate can accumulate at high concentrations, known to promote intracellular fatty acid synthesis, while also stimulating fatty acid synthesis by increasing the activity of acetyl-CoA carboxylase (ACC). Additionally, lactate can induce the upregulation of the lactate transporter SLC5A12 on CD4^+^ T cells, thus forming a positive feedback loop to increase fatty acid synthesis [36]. However, during inflammatory stress responses, acetyl-CoA generated from fatty acid oxidation can promote glycolysis, thereby enhancing lactate production, implying that lactate has an inhibitory effect on fatty acid oxidation [37–39]. Lastly, as an end-product of metabolism, lactate can serve as a substrate in post-translational protein modifications, such as lactylation and pyruvylation. Numerous studies have indicated that histone lactylation accumulates on the gene promoters of cells stimulated by hypoxia, IFN-γ, LPS, and other factors, thereby directly regulating gene expression [40, 41]. In conclusion, lactate, a key product of glycolysis, not only serves as a critical energy source for cells but also plays a crucial role in signaling transduction and gene expression regulation.

Although the production and mechanisms of lactate have been extensively studied, lactylation as a novel lactate-induced post-translational modification was identified through mass spectrometry in 2019. This modification provides a promising therapeutic target for cancer treatment. Notably, histone lactylation links metabolism, transcription, and epigenetics, regulating gene expression and influencing cell fate at the chromosomal level. The glycolytic pathway is closely associated with lactylation, and factors affecting glycolysis may also regulate this modification. Research indicates a positive correlation between high expression of SLC2A1 and CD44. Upregulation of GLUT1 promotes the polarization of tumor-associated macrophages and is associated with elevated protein lactylation levels in tumors [42]. This lactylation promotes cancer cell survival and immune evasion by regulating glycolysis in the tumor microenvironment. In pulmonary arterial hypertension, hypoxia-induced mROS inhibit HIF-1α hydroxylation via the HIF-1α/PDK1/PDK2/p-PDH-E1α axis, triggering glycolytic shift in pulmonary artery smooth muscle cells (PASMCs), leading to lactate accumulation and histone lactylation. This enhances the activity of HIF-1α target genes such as Bmp5, Trpc5, and Kit, promoting PASMC proliferation [43]. Additionally, HIF1α lactylation enhances KIAA1199 transcription, facilitating angiogenesis and tumor vasculogenic mimicry [44]. The B-cell adaptor PI3K (BCAP) regulates the transition of inflammatory macrophages to reparative macrophages by promoting histone lactylation. BCAP deficiency impedes the inactivation of FOXO1 and GSK3β, leading to enhanced inflammatory state and impaired aerobic glycolysis, and reduced lactate production [41]. PFKFB3 mediates NF-κB family activation through histone lactylation, particularly H4K12la, enriching promoters of NF-κB signaling genes such as Ikbkb, Rela, and Relb, thus activating their transcription and promoting inflammation [45].

CD44, a cell surface glycoprotein, plays a crucial role in tumor cell adhesion, migration, and survival. A shikonin-loaded system targeting CD44 demonstrates the potential to inhibit tumor growth and liver metastasis by reprogramming immunometabolism through PKM2 inhibition [46]. Furthermore, PFKFB3 regulates cancer stemness in small cell lung cancer via the Hippo pathway, and its inhibition affects cancer stemness markers like CD44, associated with chemotherapy resistance [47]. MDR1 is commonly associated with chemoresistance in tumor cells. Metastasis is linked to the metabolic characteristics of cancer stem cells (CSCs), where MDR1 + CSC subsets exhibit specific metabolic and stemness features, suggesting MDR1’s role in CSC metabolism and metastasis [48]. TFEB, a master regulator of lysosomal biogenesis and function, also regulates mitochondrial function. HKDC1, a direct target of TFEB, plays a role in glycolysis. The TFEB-HKDC1 axis is critical for PINK1/Parkin-dependent mitophagy [49]. Hence, dysregulation of the glycolytic pathway is closely associated with chemoresistance, indicating that targeting glycolysis and lactylation mechanisms could be effective in developing combination therapies. Tumor-derived lactate promotes RUBCNL expression, enhancing autophagy and resistance to Bevacizumab treatment [50]. Highly resistant bladder cancer cell subsets are associated with glycolytic metabolism, and histone H3K18la plays a crucial role in activating target gene transcription. Targeting H3K18la restored cisplatin sensitivity in these resistant cells. Additionally, transcription factors YBX1 and YY1, driven by H3K18la, play roles in promoting cisplatin resistance in tumors [51]. Further studies suggest that lactylation not only affects the phenotype of cancer cells but also promotes resistance formation by altering cellular metabolic states. For instance, Numb/Parkin-mediated mitochondrial health determines cancer cell fate through histone lactylation regulation. The Numb/Parkin-guided mitochondrial adaptation serves as a key metabolic switch for cancer cell plasticity and emerges as a promising target for cancer therapy, emphasizing the link between mitochondrial function and tumor cell resistance [52].

### Characteristics **of lactylation**

Lysine lactylation of histones, first reported by Zhang et al. in 2019, represents a novel post-translational modification of histone proteins [40]. The study identified a mass shift of 72.021 Daltons on lysine residues in three protein hydrolysates using liquid chromatography-tandem mass spectrometry analysis. This mass shift was found to be caused by the addition of a lactyl group to the ε-amino group of lysine residues. Furthermore, they identified 28 lactylation sites on histones in human and mouse cells, confirming the presence of this modification. In summary, this study established a novel histone modification regulated by lactate and demonstrated that lysine lactylation is regulated by the dynamic changes in glucose metabolism and lactate levels.

As a recently discovered form of post-translational modification, lactylation exhibits several noteworthy characteristics: (1) The primary source of substrate for lactylation is intracellular metabolic product lactate, which sets it apart from other histone modification pathways that have been identified. Lactate is primarily produced through the glycolytic pathway, making it a plentiful substrate for lactylation in cells with high glycolytic activity, such as those found in tumors. This explains the elevated levels of lactylation detected in various tumor samples. In comparison, other histone modifications, such as acetylation, primarily rely on intracellular acetyl-CoA, where the acetyl group is transferred from acetyl-CoA to the ε-amino side chain of lysine residues in proteins [53]. Methylation, on the other hand, requires S-adenosyl-L-methionine as a methyl donor, transferring the activated methyl group from S-adenosyl-L-methionine to nitrogen, oxygen, or sulfur-containing amino acid residues. (2) Lactylation sites: Lactylation, as a newly discovered post-translational modification, is found widely on both histone and non-histone proteins. Zhang et al. reported the discovery of 28 histone lactylation sites, primarily located on H3 and H4, in human and mouse M1 macrophages in the journal “Nature” in 2019 [40]. Subsequent research has identified specific lactylation sites in different cell and tissue types. For example, Yu et al. discovered increased lactylation at the H3K9 and H3K14 sites in hepatocellular carcinoma [54]. With the advancement of mass spectrometry technology, thousands of lactylation sites on non-histone proteins have been identified. These sites are widely distributed on various functional proteins, predominantly on enzymes involved in metabolic pathways. For instance, Yang et al. reported 9275 Kla sites, with 9256 located on non-histone proteins, primarily affecting enzymes involved in metabolic pathways, including the tricarboxylic acid cycle, carbohydrate, amino acid, fatty acid, and nucleotide metabolism [55]. In the study of autophagy, Jia et al. reported lactylation-mediated Vps34 (K356 and K781) through acyltransferase KAT5 / TIP60, linking autophagy and glycolysis [56]. Additionally, Xiong et al. found that lactylation can act on the zinc finger domain of the RNA methyltransferase METTL3, influencing its catalytic activity [57]. Therefore, the lactylation sites are extensive, not limited to a specific protein category, and their target specificity is closely associated with the observed biological effects (Table 1). (3) Enzymatic dependency: Histone lactylation is regulated by modifying enzymes, rather than being a spontaneous chemical reaction [58]. Histone deacetylases HDAC1-3 and SIRT1-3 can catalyze the removal of lactyl groups, indicating de-lactylation enzymatic activity. Dong et al. discovered that the YiaC enzyme can catalyze the formation of lysine lactylation in the cell, while CobB, as a lysine de-lactylase, can remove the post-translational modification both in vivo and in vitro. This suggests that lactylation is a reversible process, with its formation and removal depending on the action of specific enzymes [58, 59]. (4) Competitive relationship with acetylation: Lactylation and acetylation can occur at the same site on histones, and they compete with each other. HDAC deacetylases can not only remove acetyl groups but also catalyze the removal of lactyl groups, indicating that both modifications can occur at the same lysine site [58]. The competition between histone lactylation and acetylation marks the levels of lactate and acetyl-CoA. Whether pyruvate is dedicated to lactate or acetyl-CoA generation as an output of glycolysis determines the cell’s fate towards malignant tumor development [60].

**Table 1:** Lactylated protein sites in different cells / tissues / species

Cell / Tissue / Species	Lactylated proteins / sites	Function	Disease	PMID
HeLa	H3K9; 18; 23; 27; 79	-	cervical cancer	PMID: 31645732
HeLa	H4K5; 8; 12; 16; 31; 77; 91	-	cervical cancer	PMID: 31645732
HeLa	H2AK11; 13; 115	-	cervical cancer	PMID: 31645732
HeLa	H2BK5; 11; 15; 16; 20; 23; 43; 85; 108; 116; 120	-	cervical cancer	PMID: 31645732
Mouse BMDM	H3K14; 18; 23; 27; 56	Glutathione metabolism; Cell adhesion; Cell migration; Wound healing; Membrane permeability	-	PMID: 31645732
Mouse BMDM	H4K8; 12; 31; 91	-	-	PMID: 31645732
Mouse BMDM	H2AK11; 115	-	-	PMID: 31645732
Mouse BMDM	H2BK5; 11; 15; 16; 20; 85; 108	-	-	PMID: 31645732
MCF-7	H3K23; H4K8; H2BK5	-	Breast cancer	PMID: 31645732
92.1 / MUM2B / OCM1	H3K18	Regulating YTHDF2 and m6A methylation to accelerate tumor progression and invasion.	Melanoma	PMID: 33726814
Treg	H3K18la	Treg cells actively absorb lactate through monocarboxylic transporter 1, promoting the translocation of NFAT1 into the nucleus, thereby enhancing the expression of PD-1 and activating immune checkpoints.	Gastric cancer / lung cancer	PMID: 35090594
liver	Fatty acid synthase K673	Inhibits fatty acid synthase activity and mediates MPC1 to downregulate hepatic lipid accumulation.	Nonalcoholic fatty liver disease.	PMID: 36651176
Mouse BMDM	METTL3	Lactylation-driven METTL3 - JAK1 - STAT3 regulatory axis effectively induces the immunosuppressive function of TIMS.	Colon cancer	PMID: 35320754
Treg	MOESIN	Lactate regulates the generation of Treg cells through the emulsification of Lys72 in MOESIN, thereby improving the interaction of MOESIN with TGF-β receptor I and downstream SMAD3 signaling.	Hepatocellular carcinoma	PMID: 35732125
H1299	Vps34	Lactate connects autophagy and glycolysis through Vps34 lactylation (at lysine-356 and lysine-781), which is mediated by the acyltransferase KAT5/TIP60.	Cellular autophagy	PMID: 37267363
Hep3B / HCCLM3	H3K9; 14	Inhibits HCC development by interfering with lactate production and inhibiting H3 histone lactylation at H3K9la and H3K14la sites.	Hepatocellular carcinoma	PMID: 37453194
HCC tissue	9275 Kla sites, 9256 of which are on non-histone proteins	Lactylation of K28 inhibits the function of adenylate kinase 2 and promotes HCC cell proliferation and metastasis.	Hepatocellular carcinoma	PMID: 36593272
AGS	2375 Kla sites in 1014 proteins	Lactate affects RNA splicing in gastric cancer AGS cells.	Gastric cancer	PMID: 35800753
Oryza sativa	638 Kla sites in 342 proteins	Lysine lactylation is enriched in proteins related to central carbon metabolism and protein biosynthesis.	-	PMID: 34264677
Botrytis cinerea	273 Kla sites identified in 166 proteins	host adherence, signal transduction, primary nutrients transduction, molecular chaperons function, and ribosomal translation.	Gray mold disease	PMID: 33193272
Homo sapiens	ALDOA-K147; DHRS7-K321	-	-	PMID: 35761067
Homo sapiens	ALDOA; FPB; PGK1; DHRS7; HDAC3; ENO1	-	-	PMID: 35761068
*Escherichia coli*	1047 Kla sites in 478 proteins	Regulate bacterial metabolism.	-	PMID: 36333310
Lung tissue	724 Kla sites in 451 proteins	-	-	PMID: 37170646

### The relationship between lactylation and other post-translational modifications

Post-translational modification is a crucial step in cellular signal transduction, where specific chemical groups are transferred from one protein to another within the cell. Apart from lactylation, post-translational modifications include classical modifications such as phosphorylation, ubiquitination, SUMOylation, methylation, glycosylation, acetylation, succinylation, and malonylation. Lactylation is intricately linked with other well-established and extensively studied types of modifications.

Firstly, several studies have indicated a high degree of co-localization between histone acetylation and lactylation in the genome, suggesting potential crosstalk between lactylation and acetylation [40, 61]. Common deacetylases such as HDAC1-3 and SIRT1-3 also possess delactylation capabilities, with HDAC3 serving as an effective “eraser” of lactylation [58]. Additionally, HDAC3 is involved in the removal of lysine crotonylation and β-hydroxybutyrylation modifications [62, 63]. Macrophages uptake extracellular lactate, which promotes HMGB1 lactylation through a p300 / CBP-dependent mechanism. Simultaneously, it stimulates HMGB1 acetylation by inhibiting the deacetylase SIRT1 through the Hippo / YAP pathway and by activating the acetyltransferase p300 / CBP through β-arrestin2. These findings suggest that there are common targets for both lactylation and acetylation modifications of HMGB1 [64]. Hyunsoo et al. found that hexokinase 2 induces activation of hepatic stellate cells through histone lactylation rather than histone acetylation, with histone deacetylase inhibitors impeding HSC activation, illustrating a competitive relationship between histone lactylation and acetylation [65]. The epigenetic manifestation of lactate, namely histone lactylation, shares the same “writers” (p300) and “erasers” (HDAC1-3) as histone acetylation. This suggests a possible competition between these two epigenetic modifications in cancer cells, where lactate and acetyl-CoA can regulate separate oncogenic programs [36,851,901]. Therefore, the competitive mechanism between lactylation and acetylation can be exploited to enhance one process relative to the other. Crucially, devising specific regulatory mechanisms for this dual process is a pivotal yet unanswered question, the resolution of which could aid in the implementation of novel therapeutic approaches [66, 67].

In addition to deacetylation, lactylation also interacts with other types of modifications. In the context of melanoma research, significantly elevated levels of histone lactylation were observed in melanoma and were associated with poor prognosis. Lactylation of H3K18 positively regulates the expression of YTHDF2, which, through an m6A-dependent mRNA degradation pathway, leads to the reduction of PER1 and TP53 mRNA levels. Ultimately, this process accelerates melanoma onset [19]. The lactylation modification of H3K18 promotes the expression of METTL3 in tumor immune microenvironment cells, while also inducing lactylation of the zinc finger domain of METTL3. This process enhances the mRNA methylation modification of JAK1 and its binding with YTHDF1 to augment translation efficiency. Consequently, this amplifies the activation of the JAK1 / STAT3 signaling pathway, leading to increased expression of downstream immunosuppressive effectors such as IL-6, IL-10, and iNOS, resulting in an immunosuppressive effect [57]. Interestingly, within this regulatory pathway, the m6A-YTHDF1 axis strengthens the activation of downstream signals, intensifying the phosphorylation level of STAT3, highlighting the intricate interplay between multiple protein modifications.

Phosphorylation is the most prevalent form of covalent modification in post-translational protein regulation, playing a vital role in both prokaryotic and eukaryotic organisms. The glycolysis process initiates with the phosphorylation of glucose substrates, and lactate serves as a key metabolite linking glycolysis and oxidative phosphorylation [68]. In a model of atherosclerotic cardiovascular disease using apolipoprotein-deficient mice, the lactylation of Mecp2 (Mecp2k271la) hinders the expression of epithelial-regulated protein by binding to chromatin. This alteration in turn affects the MAPK signaling pathway by modifying the level of phosphorylation of the epidermal growth factor receptor, leading to an impact on the expression of Vcam-1, Icam-1, Mcp-1, IL-1β, and IL-6. Ultimately, this process improves the condition of atherosclerosis [69]. In another study, TNF-α induces lactylation of Sox10 through a phosphorylation-dependent mechanism via the PI3K / AKT signal, driving the transcriptional program of vascular smooth muscle cell transdifferentiation, leading to cellular necroptosis. The interaction of G protein signaling regulator 5 with AKT blocks the PI3K / AKT signaling and the phosphorylation at S24 of Sox10, underscoring Sox10 as a potential target in inflammation-related vascular disorders [70]. Research in obesity and insulin resistance has also highlighted the similar dose-dependent presentation of protein lactylation and IRS-1 serine 636 phosphorylation following sodium lactate treatment [71]. During Staphylococcus aureus infection, methylthiomethane upregulates the glycolytic pathway in peritoneal macrophages, enhancing the expression of H3K18 lactylation-specific target genes (e.g., Arg1), promoting STAT3 phosphorylation, and decreasing the levels of M1 markers in peritoneal macrophages, suggesting its potential in the context of resistant infections and sepsis treatment [72].

After identifying 503 succinylated lysine sites in 303 proteins, Xiang et al. found significant succinylation at K222 of LDHA. Intriguingly, this modification does not affect LDHA ubiquitination but diminishes the binding of ubiquitinated LDHA with SQSTM1, thereby reducing its lysosomal degradation and promoting tumor invasion and metastasis. This highlights the interplay between lactylation, succinylation, and ubiquitination pathways [73]. In LPS-activated macrophages, succinylation of pyruvate kinase 2 at lysine residue K311 impairs glycolytic activity, promotes HIF-dependent gene transcription, and leads to the production of IL-1β [74, 75]. Additionally, histone crotonylation and lactylation play critical roles in the epigenetics of neurodevelopment [76]. Crotonylation and lactylation can occur on all core histones and share the most common modification sites with histone lysine acetylation [40, 77]. This intriguing crosstalk between protein modifications is expected to become a focal point of future research [78].

## Lactylation and tumors of the digestive system

### Esophageal cancer

Esophageal cancer usually develops in the cells of the esophageal lining and primarily includes two types: adenocarcinoma and squamous cell carcinoma. Globally, the incidence of EC is estimated at around 600,000 cases, ranking it eighth in the list of prevalent cancers, with approximately 540,000 deaths, ranking it sixth. This underscores the significant threat that esophageal cancer poses to human health [79]. The primary risk factors for esophageal cancer include genetics, lifestyle habits, dietary patterns, alcohol consumption, smoking, and environmental characteristics [80]. Elevated lactate can lead to an acidic TME, thereby stimulating the progression of EC. Lactate metabolism is closely associated with the prognosis of EC [81, 82]. Overexpression of LDHA in EC tissues leads to excessive lactate production and accumulation. Reprogrammed glycolysis provides tumor cells with the rapid proliferation required energy and carbon sources, while the produced lactate participates in TME acidification, promotes angiogenesis, and downregulates immune cell activity [81]. Deng et al. found that ginsenoside Rh4 inhibits aerobic glycolysis in EC cells by hindering lactate generation, glucose uptake, and ATP production, exhibiting excellent anti-tumor effects [83]. Zheng et al. identified that the antipsychotic drug penfluridol, used for treating schizophrenia, inhibits glycolysis in EC and induces cell apoptosis, with the key enzyme PFKL in glycolysis being a direct target of penfluridol [84]. Metabolomic analysis of EC tissues revealed significant metabolic differences between tumor and non-tumor tissues, with elevated levels of lactate and citrate in tumor tissues, confirming the “Warburg” effect in tumors. Apart from glutamine, most amino acid concentrations were significantly elevated in tumor tissues, which may be related to excessive glutamine decomposition during tumorigenesis [85]. Silencing the Caprin-1 gene can inhibit EC cell proliferation and glycolysis, as well as reduce the expression of METTL3 and the tumor-related protein WTAP, thereby, affecting tumor growth [86]. Similarly, inhibiting lactate transport also demonstrates anti-tumor effects, as blocking monocarboxylate transporter increases the apoptotic levels of EC cells and reduces their proliferative capacity [87]. Thus, targeted regulation of lactate metabolism or protein lactylation processes may become a new strategy for the treatment of EC. For example, LDHA inhibitors can reduce EC cell proliferation, indicating that inhibiting the glycolysis-lactate pathway may have therapeutic effects [88]. Overall, a comprehensive analysis of the relationship between lactate and EC may provide new therapeutic ideas and targets for this disease. However, currently, there are no reports on the relationship between lactylation and EC. Subsequent research may directly explore its molecular mechanism from the perspective of epigenetics.

### Gastric cancer

Gastric cancer refers to a malignant tumor that originates from the epithelial cells of the gastric mucosa, ranking among the top five in terms of its incidence and mortality among various types of tumors [89]. According to statistics, there are approximately 1.2 million new cases of GC diagnosed globally each year, with China accounting for approximately 40%. However, the proportion of early-stage GC is relatively low, at only about 20%, with the majority of cases being diagnosed at an advanced stage. Consequently, the overall 5-year survival rate of GC is approximately 20% [90, 91]. The occurrence of GC is influenced by various factors, including *Helicobacter pylori* infection, smoking, alcohol consumption, obesity, and a high-salt diet [92]. These risk factors are particularly prevalent among GC patients in East and Southeast Asia, while the prevalence of GC in regions such as North America is relatively low [93, 94]. In recent years, in-depth research into the pathogenesis of GC has increasingly revealed the significant role of tumor metabolic reprogramming in its occurrence and development. Metabolomic analysis has discovered a significant increase in glycolytic intermediates such as lactate and pyruvate in GC tissues, with marked differences compared to adjacent normal tissues [95]. Furthermore, the myo-inositol metabolism related to fatty acid synthesis in GC tissues is found to be in an activated state, indicating a dual enhancement feature of glycolysis and lipid synthesis in GC metabolism. The dependence of tumor cells on glycolysis determines their sensitivity to glycolytic inhibitors. Maruyama et al. found that glycolytic inhibitors such as 2-DG can significantly inhibit the proliferation of GC cells and enhance chemosensitivity [96].

Recent research has revealed the crucial role of lactylation in the development of GC. Through the bioinformatics techniques, Yang et al. compared normal and tumor tissues, revealing the overexpression of four pathways closely associated with lactylation in GC tissues. Additionally, there exists a significant correlation between lactylation scores and the overall survival rate and disease progression of GC patients. GCs with high lactylation scores demonstrate increased immune cell infiltration, elevated genetic instability, and diminished response to immune checkpoint inhibitors [97]. Furthermore, a study comprehensively analyzed the lactylated proteome of GC AGS cells using mass spectrometry, which identified 2375 lysine lactylation sites on 1014 proteins. These proteins are primarily involved in spliceosomal functions. Notably, the lactylation levels in GC tissues were markedly higher than in adjacent tissues, and poor prognosis was associated with high lactylation levels. These findings not only expand the dataset of lactylated proteomes in GC but also suggest lactylation as a potential prognostic marker for GC [98]. To overcome the limitations in identifying lactylation sites using mass spectrometry, Lai et al. developed the computational model Auto-Kla using automated machine learning methods to predict protein lactylation sites. This approach is expected to become a practical tool for predicting post-translational modification sites and to provide a reference for the development of related models [99].

Lactylation scores have been strongly correlated with overall survival and tumor progression in GC patients. Specifically, patients with high lactylation scores exhibit extensive immune cell infiltration, particularly with a high degree of macrophage infiltration, which is accompanied by increased genomic instability. These characteristics suggest that tumors with high lactylation scores have a heightened risk of immune evasion and functional impairment [97]. Furthermore, immune checkpoint inhibitors (ICIs) have shown lower response rates in GC patients with high lactylation scores [97]. This indicates that alternative treatment strategies or adjustments in the use of ICIs may be necessary for patients with high lactylation scores. For instance, reducing lactylation levels to enhance tumor sensitivity to ICIs could be a potential therapeutic direction.

### Colorectal cancer

Colorectal cancer is the third most common malignant tumor globally and the second leading cause of death [100]. According to statistics from the International Agency for Research on Cancer (IARC) of the World Health Organization, the global incidence of new cases of colon cancer reached 1.9316 million in 2020, accounting for 9.7% of all malignant tumors [101]. Among them, the number of new cases of CRC in China exceeded 550,000, accounting for 28.8% of the global new cases of CRC, with more than 280,000 deaths, representing 30.6% of the global deaths from CRC [102]. Although genetic factors play a major role in the incidence of CRC, the majority of cases are not hereditary [103]. The incidence of CRC varies worldwide, with higher rates in Australia, New Zealand, Europe, and North America, and lower rates in Africa, Central Asia, and South Asia [104]. This difference may be related to dietary structure, environmental exposure, socioeconomic status, and the CRC screening rate [105].

Recent studies have found that lactylation plays a crucial role in the occurrence and development of CRC. Under hypoxic conditions, the expression level of β-catenin and the degree of lactylation in CRC cells significantly increase [106]. Tumor-produced lactate can serve as a lactylation substrate to increase lactylation of histone H3K18, indicating that the enhanced lactate production from increased glycolysis under hypoxic conditions can promote the malignant progression of tumor cells [107]. Moreover, enhanced lactylation can affect the activation of signaling pathways, thereby promoting the progression of CRC. For instance, in clear cell renal cell carcinoma, inactivation of VHL can trigger histone lactylation, activating the PDGFRβ signaling pathway, forming a positive feedback loop, and promoting tumor progression [20]. In addition to its significant role in tumor progression, lactylation is also associated with the chemoresistance of tumor cells. Studies have shown that tumor-produced lactate increases the expression of RUBCNL by inducing lactylation of histone H3K18, which can promote the autophagy process, making CRC cells resistant to bevacizumab [107]. Furthermore, downregulation of SMC4 can induce a state resembling quiescence in CRC cells through causing aberrant glycolysis, lactate accumulation, and histone lactylation, leading to slow proliferation and insensitivity to chemotherapy drugs [108]. Given the multiple roles of lactylation in the occurrence and development of CRC, therapeutic strategies targeting the lactylation process may provide new insights for the prevention and treatment of CRC. For example, PCSK9 can effectively control colon cancer by regulating tumor cell EMT and the PI3K / AKT signaling pathway, promoting M2 polarization phenotype in macrophages through mediating MIF and lactate levels, and targeting PCSK9 expression [109]. Simultaneously, the significant efficacy of inhibiting histone lactylation and PDGFRβ signaling has been observed in clear cell renal cell carcinoma [20]. The combined use of drugs that inhibit lactylation and autophagy can enhance the efficacy of bevacizumab in CRC [107]. Therefore, novel treatment strategies targeting lactylation warrant further exploration.

In CRC, tumor-derived lactate promotes H3K18la, thereby enhancing the expression of autophagy-enhancing protein RUBCNL, which contributes to resistance against bevacizumab treatment. Patients with CRC resistant to bevacizumab exhibit elevated levels of histone lactylation. Inhibition of histone lactylation has been shown to effectively suppress tumor formation, progression, and survival under hypoxic conditions in CRC [50]. Furthermore, β-catenin, a key protein in the Wnt signaling pathway, plays a crucial role in the progression of CRC. Hypoxia-induced β-catenin lactylation promotes the proliferation and stemness of CRC cells through the Wnt signaling pathway [106]. Additionally, ALDOB, identified as a potential regulator of the Warburg effect in CRC, enhances lactate production and secretion, thereby activating PDK1 and influencing carcinoembryonic antigen (CEA) expression. Lactate has been observed to upregulate LDHB in adjacent cells and is pivotal in modulating the ALDOB-mediated phenotype. The impact of ALDOB on CEA expression is a downstream effect of bioenergetic changes induced by ALDOB-mediated lactate secretion. Moreover, carcinoembryonic antigen-related cell adhesion molecule-6 controls the proliferation and chemoresistance of CRC cells [110]. Thus, lactylation-related genes play a significant role in the progression of CRC and may serve as novel therapeutic targets. Therapeutic strategies aimed at inhibiting histone lactylation or targeting the ALDOB/PDK1/lactate/CEACAM6 axis could potentially offer new treatment options for patients with CRC.

### Hepatocellular carcinoma

Hepatocellular carcinoma ranks as the sixth most common cancer globally and the fourth leading cause of cancer-related deaths [100]. In 2020, the worldwide incidence of HCC was 906,000 cases, with an age-standardized incidence rate of 9.5 per 100,000 [111]. It is projected that by 2040, the annual diagnoses of HCC and associated deaths globally will increase by over 55% [112]. HCC remains the main cause of death in patients with liver cirrhosis, with more than 90% of HCC cases occurring on the basis of chronic liver disease. Liver cirrhosis resulting from any etiology represents the most significant risk factor for HCC. Major risk factors for HCC include long-term alcohol consumption, diabetes or obesity-related metabolic fatty liver disease, as well as HBV or HCV infections [113, 114].

Lactylation has been extensively studied in HCC, with lactate emerging as a critical regulatory factor in the maintenance of cancer initiation, progression, and immune escape. Accumulation of lactate and enhanced lactylation in HCC contributes to the immune-suppressive characteristics of the TME [115]. Lactate metabolic crosstalk in the TME could significantly impact HCC progression, immunotherapy, and prognosis. Targeting lactate metabolism to restore host anti-tumor immune metabolic adaptability may further augment the therapeutic efficacy of cancer immune checkpoint blockade. Yang et al. identified 9275 Kla sites in HCC tissues using proteomic techniques, with 9256 sites located on non-histones, suggesting that Kla is a widespread modification beyond histones and transcriptional regulation [55]. Similarly, Hong et al. identified 2045 Kla modification sites from 960 proteins and quantitatively measured 1438 sites from 772 proteins. Several differentially expressed Kla proteins promoted the formation and metastasis of HCC [116]. These lactylated proteins are extensively involved in multiple biological processes in HCC, including glycolysis, the tricarboxylic acid cycle, mitochondrial function, and cytoskeletal reorganization, indicating Kla as one of the key mechanisms regulating metabolic reprogramming in HCC. Research by Jin et al. demonstrated that SIRT3 can inhibit the proliferative function of liver cancer cells by removing lactylation groups on the CCNE2 protein. Crystallographic studies elucidated the molecular mechanism of SIRT3-mediated de-lactylation of CCNE2 at the K348 site. Additionally, Honokiol promotes its anti-HCC effects by activating SIRT3 to facilitate de-lactylation of CCNE2 [117]. The expression of lactylation-related genes in HCC patients is closely associated with prognosis. Cheng et al. established a model containing eight lactylation-related genes through LASSO regression analysis, effectively predicting the prognosis of liver cancer patients. High-risk group patients distinguished by lactylation-related genes exhibit fewer tumor-infiltrating immune cells. The low-risk group shows greater sensitivity to tumor immunotherapy and a better response to immune checkpoint inhibitors, while the high-risk group is more sensitive to drugs such as sorafenib [118]. Moreover, tumor-produced lactate can enhance the immunosuppressive function of Treg cells by modifying the Moesin protein in Treg cells, enhancing its interaction with the TGF-β signaling pathway, thereby facilitating immune escape in tumors [119]. Certain natural drug components can exert anti-HCC effects by inhibiting lactate production and histone lactylation modification. For example, demethylzeylasteral (DML) can reduce intracellular lactate production by inhibiting the glycolysis pathway and suppress lactylation modifications at the H3K9 and H3K56 sites. Both of these histone modifications are closely associated with the occurrence and development of HCC. In a nude mouse liver cancer model, the inhibitory effect of DML on cancer growth through the regulation of H3 lactylation has been confirmed [120]. Additionally, royal jelly acid can specifically inhibit lactylation at the H3K9 and H3K14 sites, demonstrating its role in inhibiting the proliferation, metastasis, and apoptosis of liver cancer cells [54]. Therefore, the accumulation of lactate in the TME and protein lactylation modification may represent a crucial mechanism for tumor-induced immune suppression. The development of drugs targeting lactate metabolism and lactylation modification is expected to enhance the efficacy of HCC immunotherapy.

The expression levels of lactylation-related genes in HCC are closely associated with tumor progression. Studies analyzing gene expression data from the TCGA database have identified 16 lactylation-related genes correlated with prognosis and constructed an 8-gene signature capable of predicting clinical outcomes in HCC patients. Patients with higher risk scores generally exhibit poorer prognoses and significant differences in immune cell abundance. Additionally, patients with higher risk scores show increased sensitivity to most chemotherapeutic agents and sorafenib, whereas those with lower risk scores are more responsive to certain targeted drugs such as lapatinib and FH535 [118]. Demethylzeylasteral DML effectively inhibits tumorigenesis in liver cancer stem cells by suppressing histone H3 lactylation, particularly at H3K9la and H3K56la sites, providing new potential therapeutic targets for HCC [120]. Integrative analyses have established a novel prognostic signature based on lactylation-related genes EP300 and HDAC1-3 to predict HCC patient outcomes. The expression levels of HDAC1 and HDAC2 are significantly associated with the proportion of immune cell infiltration, especially B cells. Upregulation of HDAC1 and HDAC2 is markedly correlated with poorer prognoses, suggesting their potential as new biomarkers for HCC treatment [121]. Knockdown of Glypican-3 (GPC3) inhibits the growth, stemness, and glycolytic development of HCC cells in the hypoxic microenvironment by reducing lactylation modifications, indicating that GPC3-mediated lactylation may represent a novel therapeutic direction for HCC [122]. Furthermore, lactate and lactylation enhance immune suppression within the tumor microenvironment by mediating immune cell reprogramming and cellular plasticity, offering new perspectives for immunotherapy [115]. Investigating the functions and mechanisms of lactylation-related genes can provide new strategies and targets for clinical treatment of HCC.

### Pancreatic cancer

Pancreatic cancer is one of the most aggressive cancers with the highest fatality rate [123]. According to the GLOBOCAN estimate in 2020, the number of deaths is almost equal to the number of new cases (approximately 490,000 cases) [111]. About 90% of PC is described as pancreatic ductal adenocarcinoma (PDAC). Risk factors associated with PC include familial risk due to susceptible gene mutations, chronic pancreatitis, pancreatic cysts, and diabetes [124]. Approximately 80% of patients present with advanced or metastatic disease, usually precluding the opportunity for curative surgery. The combined 5-year survival rate for all stages is only 10%, and even early-stage patients meeting the criteria have a 5-year survival rate of less than 31% [125]. Research progress in PC lags far behind other cancers, and early clinical trials of immune therapy with anti-CTLA-4 and anti-PD-L1 antibodies have shown poor efficacy in advanced PC patients [126]. Although recent observations of regimens based on immune checkpoint stimulators / antagonists have shown promising results in patients with metastatic PC, the limitations of current immunotherapy for PC cannot be ignored [127].

Recent studies have found that lactate plays an important role in the signaling and epigenetic regulation of PC through pathways such as lactylation. Specifically, an upregulation of LDHA expression and a glycolysis positive feedback loop have been identified in PC through NUSAP1 [128]. NUSAP1, a microtubule-associated protein, can bind to c-Myc and HIF-1α, acting on the LDHA gene promoter region to upregulate LDHA expression. LDHA, as a rate-limiting enzyme, promotes glycolysis, generating lactate. Lactate, in turn, can stabilize NUSAP1 by inducing lactylation, reducing its degradation. This forms a NUSAP1-LDHA-glycolysis-lactate positive feedback loop, continuously driving glycolysis and lactate production, which may be one of the molecular mechanisms of the Warburg effect. Lactate can also induce lactylation of histones, altering chromatin structure and regulating the expression of PC-related genes [129]. As a highly locally enriched metabolic product, lactate exhibits significant differences in concentration at different sites. Regions with higher lactate concentrations exhibit higher degrees of lactylation, resulting in an open chromatin structure and upregulated expression of tumor-related genes. The degree of lactylation is positively correlated with local lactate concentration, thereby linking metabolic reprogramming and epigenetic regulation. In conclusion, lactate serves not only as an energy source for the rapid proliferation of PC cells but also as an important signaling molecule involved in the development of PC. Tumor cells satisfy their energy needs for rapid proliferation by upregulating glycolysis and lactate production, and the produced lactate serves as a signaling molecule, continuously driving the Warburg effect by inducing lactylation of key proteins such as NUSAP1. Lactate also alters epigenetics by inducing lactylation, regulating the expression of tumor-related genes. Therefore, targeting the lactate metabolism and signaling pathways of tumor cells may be an effective strategy for PC treatment, such as targeting NUSAP1 or inhibiting LDHA [129]. Further research on the role of lactate metabolism and signaling pathways in the mechanism of PC occurrence will help discover new treatment strategies.

The expression of lactylation-related genes (LRGs) has been closely associated with the proliferation and migration abilities of PC. Through the analysis of RNA sequencing and clinical data, ten differentially expressed LRGs with prognostic value have been successfully identified. These genes include SLC16A1, HLA-DRB1, KCNN4, KIF23, and HPDL, which are closely related to the OS of PC patients. Additionally, the clinical significance of LRGs in PC has been assessed by comprehensively examining infiltrating immune cells and tumor mutation burden across different subgroups. Notably, the SLC16A1 gene plays a critical role in the process of lactylation. A study has shown that SLC16A1 regulates lactylation levels in PC cells through lactate transport. Both in vivo and in vitro experiments have demonstrated that reducing the levels of SLC16A1 and its associated lactylation significantly inhibits tumor progression [130]. This suggests that targeting the SLC16A1/lactylation signaling pathway may be an effective therapeutic strategy against PC.

### Gallbladder cancer

Gallbladder cancer (GBC) is the most common cancer in the biliary tract and the sixth most common gastrointestinal malignancy [131]. The incidence of GBC exhibits regional variations worldwide, with a higher prevalence reported in specific regions of developing countries, while it is less common in developed countries, such as in Chile, India, some Asian countries, Eastern Europe, and Latin American countries, reporting more cases annually than other parts of the world [132]. This geographical distribution pattern may be attributed to differences in genetic susceptibility [133]. The prognosis of GBC is very poor, and surgical resection is currently the only curative method for localized GBC, but the recurrence rate can still be as high as 65% [134]. Additionally, the population eligible for surgery is limited to a significant extent, as over 70–90% of patients can only receive non-surgical treatments [133, 135, 136]. Due to enhanced protective effects against carcinogenesis in clinical practice, the occurrence of this disease accounts for only 1.2% of all diagnosed cancers worldwide.

Currently, there are no reports linking GBC to lactylation. However, a notable correlation has been found between GBC and glycolysis-related proteins (HR: 2.16, 95% CI: 1.70–2.75) [137]. Multiple research findings support the existence of glycolytic reprogramming in GBC, with increased levels of LDH and lactate correlating with tumor progression and poor prognosis. Elevated expression of key glycolytic enzymes such as glucose transporter GLUT1, LDHA, and PKM2, as well as increased lactate production, have been observed in GBC tissues and cell lines, indicating their reliance on glycolysis as an energy metabolism pathway [138]. Mechanistically, the glycolytic enzyme LDHA is a downstream target of miR-30d-5p, and downregulation of miR-30d-5p can release the inhibitory effect of LDHA, promoting GBC cell glycolysis and proliferation [29,569,755]. Similarly, downregulation of miR-181b-5p inhibits GBC vitality, migration, and glycolysis process by upregulating pyruvate dehydrogenase complex component X (PDHX) under hypoxia [139]. MiRNA-139-5p partially suppresses cell proliferation, migration, and glycolysis in GBC by inhibiting PKM2 [138]. Targeted therapy for tumor metabolism has become a new focus of precision medicine for tumors and is expected to become a new personalized treatment option for GBC patients. Numerous clinical retrospective studies have found that elevated levels of serum and bile lactate concentration and tumor LDHA expression are closely related to the pathological grading, clinical staging, and prognosis of GBC [140, 141]. This finding suggests that lactate metabolism markers may serve as new biological indicators for assessing the condition of GBC and predicting prognosis.

## Molecular mechanism

### Signaling pathways related to lactylation regulation

Glucose serves as the primary source of intracellular lactate. Glycolysis induces lactate production and regulates protein Kla levels [40]. Kla widely influences the activity and flux of various metabolism-related pathways, playing a significant role in cellular metabolic regulation. It involves lactate participating as a substrate in histone protein modifications, thereby influencing cellular metabolic pathways. Lactate concentration directly impacts lactylation levels, hence the regulation of metabolic pathways is closely associated with lactylation [142]. In tumor-related studies, Kla has been found to be closely associated with metabolic reprogramming of cancer cells and the growth and proliferation of tumor cells. Tumor cells often exhibit characteristics of metabolic reprogramming, including alterations in glucose, lipid, and amino acid metabolic pathways, with Kla modifications playing a key role in regulating these processes [143]. For instance, in HCC, enhanced glycolysis and elevated lactate concentration are important features [54]. Kla primarily affects enzymes involved in metabolic pathways, including the tricarboxylic acid cycle, as well as carbohydrate, amino acid, fatty acid, and nucleotide metabolism [55]. Kla-related models have demonstrated robust predictive efficiency for HCC, with significant enrichment of the glycolytic pathway in HCC tumor samples [118]. Demethylzeylasteral inhibits the progression of HCC, while increased H3 histone Kla effectively promotes HCC progression, suggesting the detrimental role of Kla in the progression of HCC [120]. Similarly, Royal jelly acid suppresses the development of HCC by interfering with lactate production and inhibiting histone Kla [54]. Furthermore, in CRC research, GPR37 promotes LDHA expression and glycolysis, leading to an increase in H3K18la Kla, resulting in upregulation of CXCL1 and CXCL5 [144]. Overall, the relationship between Kla and metabolic pathways has been well-established in multiple biological processes. Kla, as an important form of protein modification, plays a crucial role in cellular metabolic regulation.

**Fig. 2:**
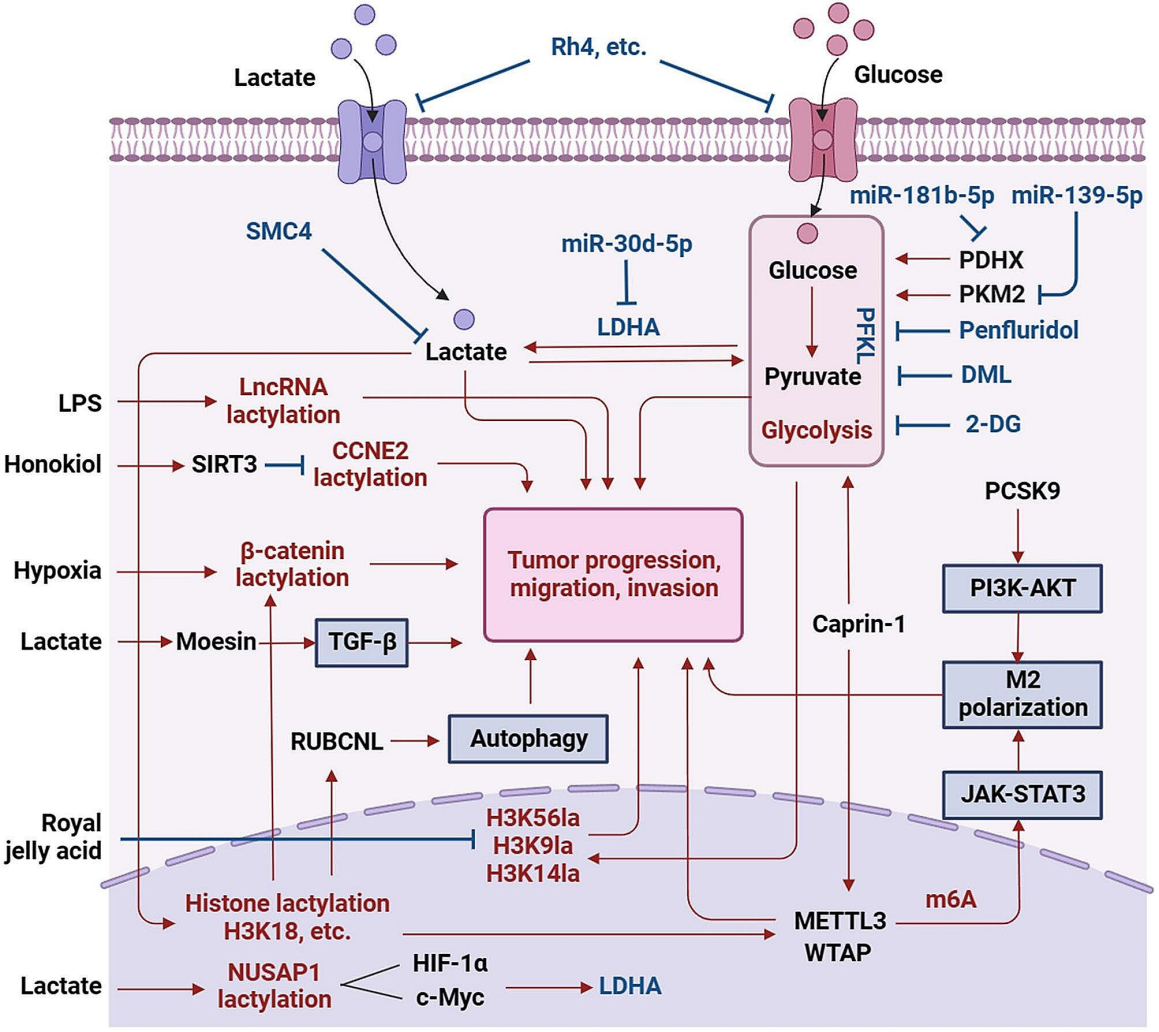
Lactate metabolism, lactylation and digestive system tumors. In tumors of the digestive system, lactate-mediated lactylation through the glycolytic pathway is regulated by various mechanisms. This regulation promotes tumor initiation, proliferation, migration, and invasion. Some substances, such as Rh4, inhibit glycolysis by reducing lactate production and glucose uptake, which demonstrates anti-tumor effects. Other substances like Penfluridol, DML, 2-DG, miR-30d-5p, miR-181b-5p, and miR-139-5p suppress tumor progression by inhibiting the glycolytic pathway. Additionally, glycolysis and lactate-mediated histone lactylation (such as H3K18la, H3K56la, H3K9la, H3K14la) play a role in tumor initiation. Histone lactylation modification also increases the expression of METTL3, which, through mediating RNA m6A modification, enhances the translation efficiency of JAK1, thereby activating the JAK1/STAT3 pathway. This transformation induces M2 polarization of tumor-associated macrophages, which suppresses the anti-tumor immune response. The PCSK9-activated PI3K/AKT pathway exhibits similar effects. Histone lactylation also promotes RUBCNL-mediated autophagy, which enhances tumor cell resistance. Both histone and non-histone lactylation levels are induced by LPS and hypoxia treatment, promoting tumor cell migration and invasion. Royal jelly acid specifically inhibits lactylation at H3K9 and H3K14 sites, demonstrating its role in inhibiting tumor proliferation, migration, and apoptosis. Lactate can also modify the Moesin protein, enhancing its interaction with the TGF-β signaling pathway. NUSAP1, a microtubule-associated protein, binds to c-Myc and HIF-1α, acting on the LDHA gene promoter region to upregulate LDHA expression. LDHA, as a rate-limiting enzyme, promotes glycolysis and lactate production. Lactate, in turn, stabilizes NUSAP1 protein through the induction of lactylation, reducing its degradation. m6A: N6-methyladenosine. PDHX: Pyruvate dehydrogenase complex component X. PKM2: Pyruvate kinase M2. PFKL: Phosphofructokinase, liver type. 2-DG: 2-deoxy-d-glucose. LDHA: Lactate dehydrogenase A. Rh4: a Ginsenoside. The figure was created using Biorender.com

Kla plays a significant role in regulating the activity of immune cells, inflammatory responses, and interactions between immune cells. Kla modifications can affect the activity of specific inflammatory signaling pathways and the interaction patterns between immune cells, thereby regulating the intensity of inflammatory responses, the formation of immune cell clusters, and the coordination of immune reactions. PCSK9 is highly expressed in colon cancer tissue, inducing the EMT process of colon cancer cells and activating the PI3K / AKT signaling, while suppressing PCSK9 expression promotes M1 macrophage polarization by reducing lactate, protein Kla, and macrophage migration inhibitory factor. This has an impact on the proliferation, migration, and invasion of tumor tissue [109]. The transition of reparative macrophages is determined by the B-cell adapter of PI3K (BCAP), and the lack of BCAP hinders the deactivation of FOXO1 and GSK3β, leading to enhanced inflammatory states and defects in aerobic glycolysis and reduced lactate production, which translates into reduced histone Kla and reduced repair macrophage gene expression [41]. Tumor cells treated with PI3K inhibitors reduce lactate production and inhibit histone Kla (H3K18lac) within TAMs, resulting in their activation as anti-cancer phagocytes [21]. Hypoxia-induced glycolysis significantly increases the Kla level of β-catenin in CRC cells, promoting tumor cell proliferation and stemness through the Wnt signaling pathway [106]. Furthermore, tumor-derived lactate promotes autophagy through H3K18lac-enhanced RUBCNL expression, thereby promoting resistance. CRC patients resistant to bevacizumab treatment exhibit elevated levels of histone Kla, and inhibiting histone Kla effectively suppresses the occurrence, progression, and survival of CRC tumors. Histone Kla promotes the transcription of RUBCNL / Pacer, promotes autophagosome maturation by interacting with BECN1 (beclin 1), and mediates the recruitment and function of the class III phosphatidylinositol 3-kinase complex, which plays a crucial role in hypoxic cancer cell proliferation and survival [107]. Additionally, in epigenetic mechanisms, increased expression of METTL3 is associated with poor prognosis in colon cancer patients, and the METTL3-mediated m6A-YTHDF1 axis enhances JAK1 protein translation efficiency and subsequent STAT3 phosphorylation. Accumulated lactate in the TME effectively induces upregulation of METTL3 in tumor-infiltrating myeloid cells via H3K18 Kla, and Kla modification sites have also been identified in the zinc finger domain of METTL3, which is crucial for the capture of target RNA by METTL3 [57]. The pattern diagram of lactate metabolism, lactylation and digestive system tumors is shown in Fig. 2.

### Lactylation and tumor microenvironment

**Fig. 3:**
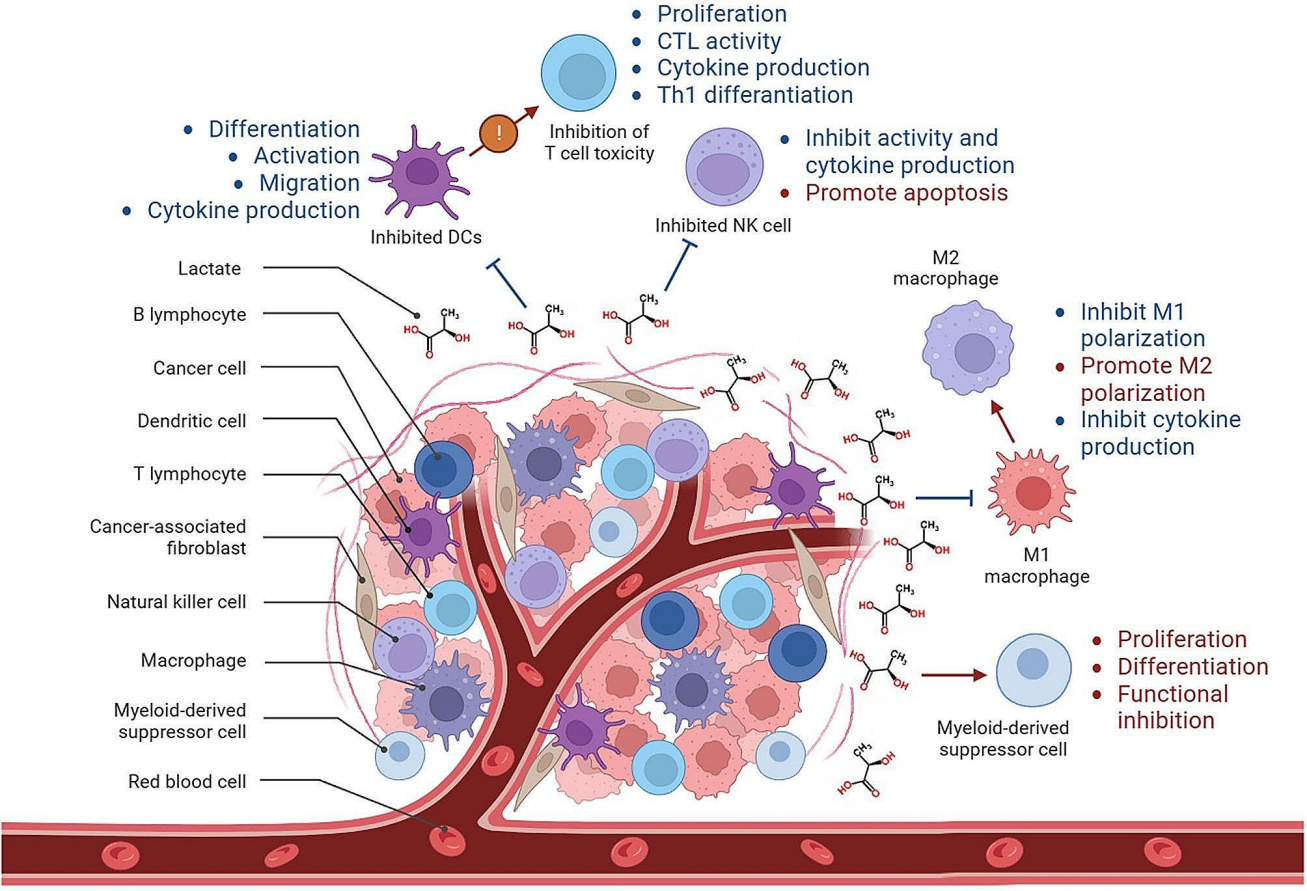
Lactate-mediated immunosuppression in the tumor microenvironment. The tumor microenvironment consists of tumor cells, tumor-associated immune cells, and tumor-associated fibroblasts, among others. In this microenvironment, lactate plays an immunosuppressive role by inducing, recruiting, and regulating immunosuppressive cells to promote tumor development. Lactate modifies histones directly, inhibiting signaling pathways. The most hypoxic regions in the tumor microenvironment typically exhibit the highest lactate concentrations, with cells relying predominantly on glycolysis as their main metabolic pathway. Lactate lowers the intracellular pH of immune cells and inhibits various immune cell activities. It suppresses the differentiation, activation, migration, and cytokine production of DCs, leading to a simultaneous inhibition of cytotoxic T cells, reducing their activation levels and cytokine production. Lactate inhibits the activity of NK cells and promotes apoptosis. It suppresses M1 polarization in macrophages while promoting their conversion to the M2 phenotype. Conversely, lactate promotes the maintenance of immunosuppressive function in MDSCs in an acidic environment, contributing to tumor occurrence and progression by regulating MSDCs. DCs: Dendritic cells. NK cells: Natural killer cells. MSDCs: Myeloid-derived suppressor cells. The figure was created using Biorender.com

Glycolysis generates lactate, leading to an acidic TME, which increases the complexity of cancer cell metabolic heterogeneity. Immune cell reprogramming and cellular plasticity mediated by lactate and lactylation increase immune suppression in the TME, serving as a key factor in regulating tumor development, metastasis, and the effectiveness of immune therapies such as immune checkpoint inhibitors [115, 145]. Immune checkpoint inhibitor (ICI) therapy has made significant progress in amplifying endogenous anti-tumor T cell responses. However, most cancer patients exhibit innate or acquired resistance to ICIs, with a significant reason being the complex metabolic landscape of the TME formed by tumor cell metabolic dysregulation, hypoxia, acidity, and glucose deficiency [146]. Excessive lactate contributes to the establishment of an immune-suppressive environment favoring cancer cell growth, promoting immune suppression, and facilitating tumor cell invasion and metastasis [147]. Lactate in the TME can exert immunosuppressive functions, promoting tumor development by inducing, recruiting, and regulating immune-suppressive cells (Fig. 3) [148]. Tumor cells, tumor-associated immune cells, and tumor-associated fibroblasts collectively constitute the TME. The most hypoxic parts of the TME typically contain the highest concentrations of lactate, with cells primarily relying on glycolysis as their main metabolic pathway. Increased expression of MCT4 aims to transport more lactate extracellularly. Subsequently, lactate diffuses to adjacent cells, which metabolize mainly via oxidative phosphorylation, and their expression of MCT1 increases to transport more lactate intracellularly and generate energy more efficiently. This shift in energy metabolism reprogramming is also one of the crucial driving forces shaping the TME [129]. Additionally, as a lactate receptor, GPR81 is highly expressed in tumor cells and their immune cells [149, 150]. The high lactate state inhibits immune cells and recruits other cells in various ways to drive tumor development, with Kla being one of the reasons. Kla promotes macrophage M2 polarization, transforming macrophages into an anti-inflammatory phenotype [151]. Acidification lowers the pH within immune cells and inhibits the activity of various immune cells, including T cells, NK cells, and dendritic cells. However, whether Kla affects these immune cells remains to be explored. Myeloid-derived suppressor cells (MDSCs) are a prominent population of immunosuppressive immune cells with broad immunosuppressive functions, such as restricting T cell function, proliferation, and TCR signal transduction, and promoting Tregs differentiation [152]. MDSCs gene expression is associated with glycolysis gene expression, and both are associated with reduced survival. Increased glycolysis and lactate concentration induce MDSC development and immune suppression, leading to an increase in the frequency of MDSC expansion, the suppression of NK cell function, and the restriction of innate immune effector activity. This suggests that lactate can establish a tumor immune-suppressive microenvironment, promoting tumor occurrence and development by regulating MSDCs [153]. Moreover, regulatory T (Treg) cells play a crucial role in maintaining an immune-suppressive TME. Lactate can enhance the stability and function of Treg cells, while lactate degradation can reduce Treg cell induction, increase anti-tumor immunity, and reduce tumor growth. Mechanistically, lactate regulates the generation of Treg cells by Kla of Lys72 in MOESIN, thereby improving the interaction between MOESIN and transforming growth factor-beta (TGF-β) receptor I and downstream SMAD3 signals [154]. In the TME, lactate has been found to modulate many cells closely associated with tumor development. However, the role of Kla in these cells is still unexplored. Investigating this issue may provide further insights into the role of epigenetics in shaping the TME.

## Prospects and challenges

In digestive system tumors, Kla modification, as an emerging area of research, has provided us with profound insights and understanding. This study systematically elaborates the various mechanisms of Kla modification in digestive system tumors, including its crucial role in tumor cell metabolism remodeling, the formation of immune-suppressive microenvironments, and tumor development and metastasis. These studies have revealed the close association of Kla modification with the development of digestive system tumors, providing new ideas and directions for future clinical treatments. Despite the widespread recognition of Kla as an important regulatory factor in tumor development and immunotherapy, its therapeutic potential and applications still face some challenges. Firstly, treatment strategies targeting Kla require deeper research and exploration to ensure their efficacy and safety. Particularly, a deeper understanding is needed regarding the potential toxicity to normal cells and its impact on physiological processes. Secondly, the application of Kla regulation in tumor immunotherapy requires more clinical validation and practice. Although some studies have suggested that combining Kla regulation with immunotherapy may enhance efficacy, more clinical trials and research are needed to confirm the feasibility and effectiveness of its clinical application. Additionally, the mechanisms and epigenetic regulatory networks of Kla modification in different digestive system tumors still need to be explored and understood in depth. This includes more detailed research on the specific role of Kla modification in the regulation of tumor cell metabolic pathways, immune evasion mechanisms, and its impact at different stages of digestive system tumor development. These in-depth studies will provide us with more precise therapeutic targets and personalized treatment strategies. Finally, despite the potential therapeutic effects of Kla regulation in tumor treatment, more research is needed to address the challenges and limitations it may face in clinical applications. This includes addressing issues such as the durability of treatment effects, the development of resistance, as well as potential side effects and safety concerns. Resolving these issues requires multidimensional research and collaborative efforts, including basic scientific research, clinical trials, and accumulated experience in clinical practice.

## Data Availability

No datasets were generated or analysed during the current study.
